# Trehalose Reverses Cell Malfunction in Fibroblasts from Normal and Huntington's Disease Patients Caused by Proteosome Inhibition

**DOI:** 10.1371/journal.pone.0090202

**Published:** 2014-02-25

**Authors:** Maria Angeles Fernandez-Estevez, Maria Jose Casarejos, Jose López Sendon, Juan Garcia Caldentey, Carolina Ruiz, Ana Gomez, Juan Perucho, Justo García de Yebenes, Maria Angeles Mena

**Affiliations:** 1 Department of Neurobiology, Ramón y Cajal Hospital, Madrid, Spain; 2 Department of Neurology, Ramón y Cajal Hospital, Madrid, Spain; 3 CIBERNED, Instituto de Salud Carlos III, Madrid, Spain; University of Florida, United States of America

## Abstract

Huntington's disease (HD) is a neurodegenerative disorder characterized by progressive motor, cognitive and psychiatric deficits, associated with predominant loss of striatal neurons and is caused by polyglutamine expansion in the huntingtin protein. Mutant huntingtin protein and its fragments are resistant to protein degradation and produce a blockade of the ubiquitin proteasome system (UPS). In HD models, the proteasome inhibitor epoxomicin aggravates protein accumulation and the inductor of autophagy, trehalose, diminishes it. We have investigated the effects of epoxomicin and trehalose in skin fibroblasts of control and HD patients. Untreated HD fibroblasts have increased the levels of ubiquitinized proteins and higher levels of reactive oxygen species (ROS), huntingtin and the autophagy marker LAMP2A. Baseline replication rates were higher in HD than in controls fibroblasts but that was reverted after 12 passages. Epoxomicin increases the activated caspase-3, HSP70, huntingtin, ubiquitinated proteins and ROS levels in both HD and controls. Treatment with trehalose counteracts the increase in ROS, ubiquitinated proteins, huntingtin and activated caspase-3 levels induced by epoxomicin, and also increases the LC3 levels more in HD fibroblast than controls. These results suggest that trehalose could revert protein processing abnormalities in patients with Huntington's Disease.

## Introduction

Huntington's disease is an autosomal dominant neurodegenerative disorder caused by the expansion of CAG repeats in the huntingtin gene [1]. Huntingtin protein plays an important role in synaptic function, is necessary in post-embryonary period, could have antiapoptotic effects and also a protector effect against mutant huntingtin [2]. Mutant huntingtin accumulates in cells of patients with HD, translocates to the nucleous, alters gene transcription, mitochondrial function and caspase activity and leads to cell death. Mutant huntingtin inhibits 26 S proteasoma activity [3] and modulation of autophagy counteracts it [4], The toxicity induced by mutant huntingtin, can lead to defects in RNA synthesis, cell survival, ubiquitin proteasome system (UPS) and also produces cellular inclusions, impairs mitochondrial activity and activates pro-apoptotic molecules [5]–[8].

Macroautophagy, chaperone-mediated autophagy and ubiquitin-proteasome system, all complementary, are the mayor systems to process abnormal proteins. It has been shown that blockade of UPS results in an increase in autophagy activity that could compensate the impaired UPS function [5]. Besides UPS, another important mechanism to degrade proteins is the autophagy-lysosomal pathway [9]–[12]. Both systems are implicated in degradation of abnormal proteins that accumulate in neurodegenerative diseases [10], [13]–[17].

Epoxomicin is a cell-permeable and irreversible inhibitor of proteasome activity resulting primarily in inhibition of the chymotrypsin-like activity [6]. Trehalose is a disaccharide that protects from environmental stresses by preventing protein denaturation [7], [8]. Trehalose is described as a chemical chaperone that helps in protein folding interacting directly with them [8], [9]. Recently, trehalose was shown to inhibit polyglutamine-mediated aggregation in vitro and in vivo models of Huntington disease [10], [11].

Recent studies have shown that trehalose increases the number of autophagosomes and markers of autophagy in Human neuroblastoma cells (NB69) and prevents injuries induced by the proteasome inhibitor, epoxomicin [12]. Other studies have shown that trehalose improves dopamine cell loss and tau pathology in parkin null mice expressing knocked-in human mutant tau [18].

The purpose of our study was to investigate the epoxomicin effects on protein accumulation and cell viability in skin fibroblasts of control and HD patients. Moreover, we have studied whether the stimulation of autophagy by trehalose, as a compensatory and protective mechanism against UPS dysfunction, reverts epoxomicin-induced damage.

## Materials and Methods

### Ethics statement

This work was performed with primary human cells in culture and was approved by the Ethical Committee for the Research of the Ramón y Cajal Hospital in Madrid. Written informed consent was obtained from the subjects themselves, which according to the Spanish Law of Biomedical Research 14/2007 and due to the nature of the study, is sufficient to perform the investigation.

### Skin fibroblasts cultures

Human fibroblast were obtained from skin biopsies of healthy and Huntginton's disease patients. Skin biopsies were cut up and put into a flask and grown in Amniomed medium (Genycell, EK AMG-200). After the first passage, Amniomed medium was changed to a medium containing Dulbecco's modified Eagle's medium (DMEM) with high glucose (4.5 g/l) (Biowest, L0101–500), 4 mM L-glutamine (GIBCO, 25030–024), 1 mM sodiumpyruvate (GIBCO, 11360–039), penicillin/streptomycin/ fungizone (100 U/ml) (GIBCO, 15240–062) and 15% fetal bovine serum (USA origin) (GIBCO-Life Technologies, 16000–044). For detection of ubiquitinated proteins, the medium was replaced by a defined medium MEM/Ham's F12 1∶1 (F12; PAA, E15–817, MEM; GIBCO, 041–01095), 20 nM progesterone (Sigma-Aldrich, P6149), 100 mM putrescine (Sigma-Aldrich, P5780), 30 nM sodium selenite (Sigma-Aldrich, S9133), 5 mg/ml insulin (Sigma-Aldrich, I1882) and 100 mM transferrin (Boehringuer, M1073974) supplemented with glucose 0.6% (Sigma-Aldrich G8769). Cells were maintained four days on culture before treatment with epoxomicin and trehalose.

### Chemicals

Epoxomicin (Calbiochem, 324800), Trehalose (Calbiochem, 625625), Suc-Leu-Leu-Val-Tyr-AMC (Calbiochem, 539142). The BCA protein assay kit was from Pierce (Pierce, 23228, 1859078), 3-Methyladenine (Sigma-Aldrich, M9281).

### Cell viability measurements and proliferation assay

Fibroblast survival was measured analysing the percentage of cells immunoreactive to cleaved caspase-3. Cultures were fixed with 4% paraformaldehyde, washed in 0.1 M phosphate-buffered saline, pH 7.4 (PBS), permeabilized with ethanol-acetic acid (19∶1), and incubated at 4°C for 24 h with a rabbit polyclonal anti-cleaved caspase-3 (1/400) (Cell-Signaling, 9664P). Cells were then washed ×3 in PBS and incubated with anti-mouse Alexa Fluor 488 (Green) (Invitrogen, A11034) for 1h at room temperature. After the final 3 washes with PBS, cover slips were mounted in anti-fading solution, 3×10−6 M final concentration and viewed under a fluorescent microscope. To assess antibody specificity of cleaved caspase-3, negative control was included by omission of the primary antibody.

To assay cell number and cell proliferation index, cell cultures were treated with trehalose (100 mM) for 15 min before incubating with the proteasome inhibitor epoxomicin (15 nM) for another 24 hours. Then were incubated with 50 mM BrdU (5-bromo-2′-deoxyuridine) (SIGMA-Aldrich, B-5002) for 24 h more before fixation and, for immunodetection, we used a mouse anti-BrdU antibody (1/20) (DAKO, M0744) and anti-mouse Ig-fluorescein antibody. Nuclei were stained by bis-benzimide (Hoechst33342) (SIGMA-Aldrich, B-2261) and immunostaining was visualized under fluorescent microscopy. The number of immunoreactive cells was counted in pre defined parallel strips.

### Measurement of ROS production

The abundance of reactive oxygen species (ROS) was determined by using 2′,7′-dichlorofluorescein diacetate (C-H2DCFH-DA; Invitrogen, C6827). Control and HD deficient fibroblasts were seeded at a density of 1×10^4^ cells/cm^2^ in glass cover slides pre-coated with poly-D-lysine (4.5 μg/cm^2^) (SIGMA-Aldrich, P6407-5MG) at 1×10^4^ cells/cm^2^ three days before the experiment. After 15 min of treatment with trehalose (100 mM), the cells were incubated with the proteasome inhibitor epoxomicin (15 nM) for another 24 hours. After this time, the cells were washed twice, and incubated with 5 μM DCFH-DA in MEM free of phenol red for 30 min, at 37°C in the incubator. Then, the cells were washed twice with (PBS with 1 mM glucose), and the nuclei were stained with bis-benzimide (Hoechst 33342) added to the anti-fading solution, at a 3×10M^−6^ final concentration. For quantitative determinations, the cover slides were observed under a fluorescent microscope using FITC (fluorescein isothiocyanate) filter and counted in 1/10 of the cover slide area; ROS positive cells were identified by fluorescence emission and total cells by bis-benzimide stained.

### Immunocytochemistry

We analyzed the percentage of immunoreactive cells to LC3 (microtubule-associated protein1 *light chain* 3) and to both HSC70 (heat shock cognate 71 kDa protein) and LAMP-2A (lysosome-associated membrane protein 2-A) as markers of macro-autophagy and chaperon-mediated autophagy [15]. The fibroblast cultures were fixed with 4% paraformaldehyde, washed in 0.1 M phosphate-buffered saline, pH 7.4 (PBS), permeabilized with ethanol-acetic acid (19:1), and incubated at 4°C for 24 h with primary antibodies diluted in PBS containing 10% fetal calf serum. Rabbit polyclonal anti-LC3 antibody (MBL, PM036) was diluted at 1/200. Mouse monoclonal anti-huntingtin mAB 2166 from (Chemicon, mAB 2166) was diluted 1/500. To measure huntingtin inside the fibroblasts we had to use the integrated Optical Density (IOD) as skin fibroblasts do not show huntingtin aggregates [14]. Mouse anti- HSC70 (heat-shock protein 70) diluted 1/100 (Abcam, Ab2788) and rabbit anti-LAMP-2A diluted 1/100 (Abcam, Ab37024). Fluorescein- and rhodamine-conjugated secondary antibodies were employed to visualize positive cells under fluorescent microscopy. Colocalization images for HSC70 and LAMP-2A were acquired using a Nikon C1 plus ECLIPSE Ti-e microscope. The number of immunoreactive cells was counted in one-seventh of the total area of the cover slides. The cells were counted in predefined parallel strips using a counting reticule inserted into the ocular.

### Detection of ubiquitinated proteins

Human skin fibroblasts cultures were treated with epoxomicin (15 nM) or pre-treated with trehalose (100 mM) 15 min before the treatment with epoxomicin for 24 h. Cells were washed with PBS plus phenylmethylsulfonyl fluoride (PMSF) (SIGMA-Aldrich, P7626), scraped in 150 μL of lysis buffer [50 mM Tris HCl, 150 mM NaCl, 20 mM EDTA, 1% Triton X-100, 50 mM sodium fluoride (NaF), 20 mM N-ethyl-maleimide, 100 μM sodium ortovanadate, 1 mM PMSF and protease inhibitors cocktail (Calbiochem, 539131)] and immediately boiled for 5 min. The lysates were centrifuged at 12.000×g at 4°C for 30 min. The supernatant was used for protein determination by the BCA protein assay kit. For detection of ubiquitinated proteins by western blot, 15 μg of protein were conducted to immunoblot assay with a mouse monoclonal antibody to ubiquitin diluted 1/500 (Chemicon, MAB1510). The secondary antibodies (1/1000) followed by ECL (enhanced chemiluminescence) detection reagents (Bio-Rad, 970442/3) were used for immunodetection. Immunoblot of β-actin diluted (1/5000) (SIGMA-Aldrich, A5441) was performed to demonstrate equal protein loading. The blots were quantified by computer-assisted video.

### Western blot analysis

Fibroblast cultures were homogenized with a sonicator in lysis buffer containing 20 mM Tris HCl, 10 mM potassium acetate (AcK), 1 mM dithiothreitol (DTT), 1 mM EDTA, 1 mM PMSF, 1 mM benzamidine, leupeptin, aprotinin, pepstatin 5 µg/ml each, 0.25% NP-40, pH 7.4, and then centrifuged at 12.000 g for 30 min at 4°C. For p-ERK and total ERK (microtubule-associated protein1 *light chain* 3) detection, 10 mM NaF, 2 mM sodium molibdate, 10 mM β-glicerophosphate, and 0.2 mM ortovanadate, were added to the lysis buffer. The supernatant was used for protein determination by the BCA protein assay kit and for electrophoretical separation. Samples (20–30 µg protein) were added to SDS sample loading buffer, electrophoresed in 10–15% SDS-polyacrylamide gels and then electroblotted to 0.45 µm nitrocellulose membranes, as described previously [19], [20].

The antibodies used in the study were the following: the chaperone mouse anti-HSP-70 (1/750) was from Santa Cruz (Heidelberg, Germany). Mouse monoclonal anti-huntingtin mAB 2166 (Chemicon, mAB 2166). Mouse monoclonal anti-ERK1/2 (SIGMA-Aldrich, M5670), Anti-P-ERK1/2 (SIGMA-Aldrich, M8159). Monoclonal anti- β-actin antibody diluted 1/5000 (SIGMA-Aldrich, A5441) diluted 1/10000 were used as a control of charge after inactivation of nitrocellulose membrane with sodium azide.

### Proteasomal activity measurement

After culture treatments, cells were washed with PBS, harvested in proteasome lysis buffer and lysed by sonication (VibraCell, level 0.5 for 30 s). Lysates were centrifuged at 12.000 g at 4°C for 30 min. The protein concentration was assayed from the resulting supernatants by the BCA protein assay kit. The 20 S proteasomal chymotrypsin-like activity was quantified by monitoring the accumulation of the fluorescent cleavage product 7-amino-4-methylcoumarin (AMC) from the synthetic proteasomal substrate Suc-Leu-Leu-Val-Tyr-aminomethylcoumarin (LLVY-AMC) (Calbiochem, 539142) using the 20 S proteasome activity assay kit (Chemicon) according to the manufacturer's instruction (measurement every 30 min for 3 hours).

### Statistical analysis

The results were statistically evaluated for significance with one-way ANOVA with repeated measures with two factors followed by Bonferroni multiple comparison test. The interactions between the genotype and the treatment were analyzed by two-way ANOVA followed by Bonferroni post-test. Differences were considered statistically significant when p<0.05. Analysis of data was performed using the SPSS software.

## Results

### Epoxomicin induces the apoptotic pathway in control and HD human skin fibroblasts and trehalose protects from it

**Figure 1:**
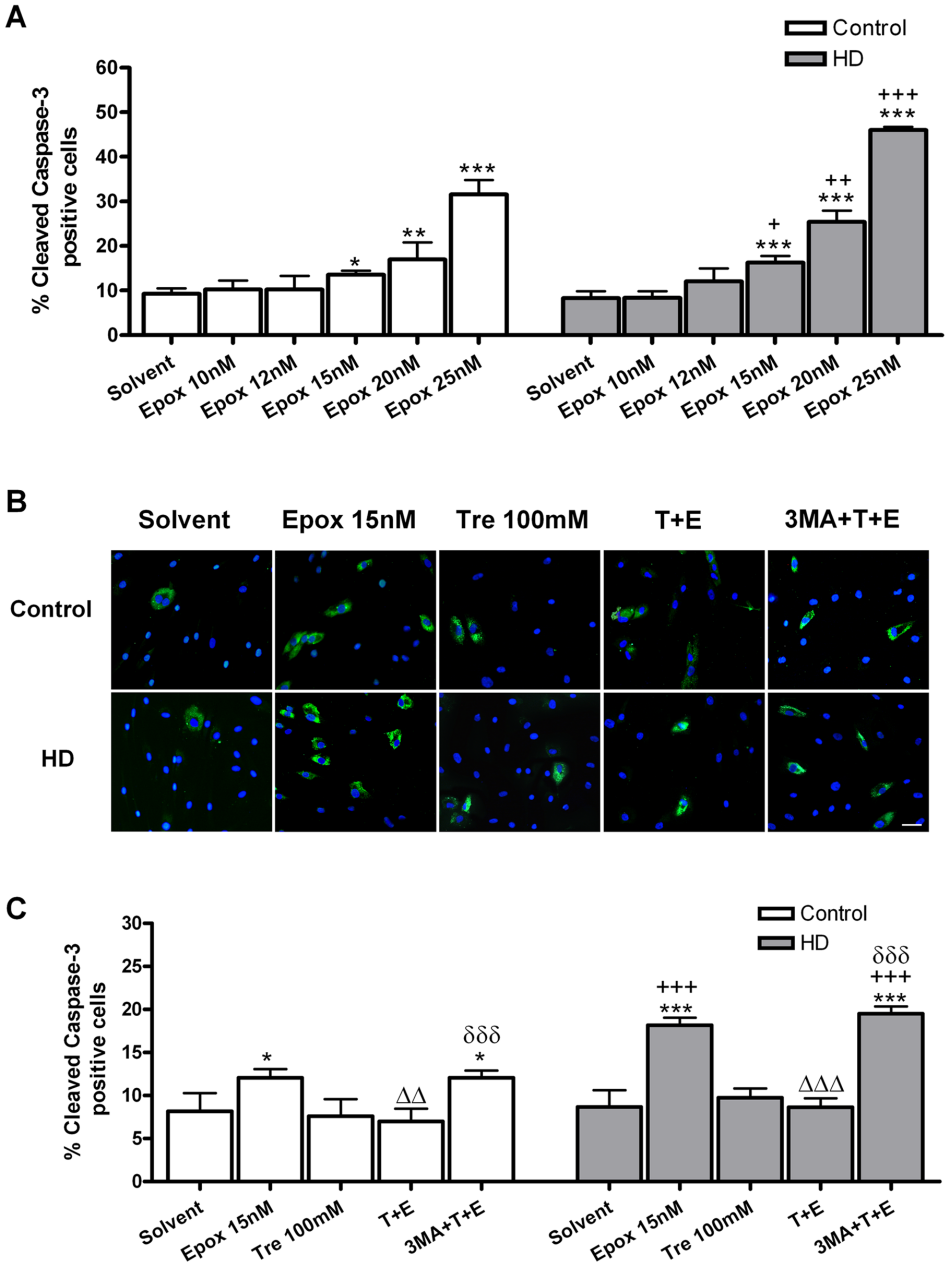
Epoxomicin and trehalose differential effects in cellular viability on control and HD human skin fibroblasts. (A) Dose-dependent effects of epoxomicin in caspase-3 activation, an indicator of apoptosis. (B) Photomicrographs of activated caspase-3^+^ cells (green) and total nuclei stained with bis-benzimide (blue) after epoxomicin and trehalose treatments. (Scale bar  = 20 µm). (C) Percent of activated caspase-3^+^ cells in control and HD fibroblasts after epoxomicin and trehalose treatments. Values are expressed as the mean ± SD, *n* = 4 patients. Control cell number (mean per field) 31.87±1.124, *n* = 4. HD cell number (mean per field) 44.75±2.456, *n* = 4. The data of each patient was obtained using 4 replicates. Statistical analysis was performed by one-way ANOVA with repeated measures followed by Bonferroni multiple comparison test: *p<0.05, **p<0.01, ***p<0.001 *vs* Solvent; +p<0.05, ++p<0.01, +++p<0.001 HD *vs* controls; ΔΔp<0.01, ΔΔΔp<0.001 trehalose + epoxomicin *vs* epoxomicin; δδδp<0.001 3-methyladenine + trehalose + epoxomicin *vs* trehalose + epoxomicin.

Epoxomicin activates, dose-dependently, the pro-apoptotic protein caspase-3. This effect is more pronounced in HD than in control fibroblasts (FIG. 1A). For the following experiments, we chose the 15 nM dose because was the minimum dose that discriminated both genotypes. After 24 hours of treatment, the effects of epoxomicin 15 nM in caspase-3 activation are reversed by trehalose 100 mM when added 15 min before epoxomicin and the protection is inhibited using the autophagy inhibitor 3-methyladenine (FIG. 1B–C).

### Trehalose increases the number of skin proliferative cells

**Figure 2:**
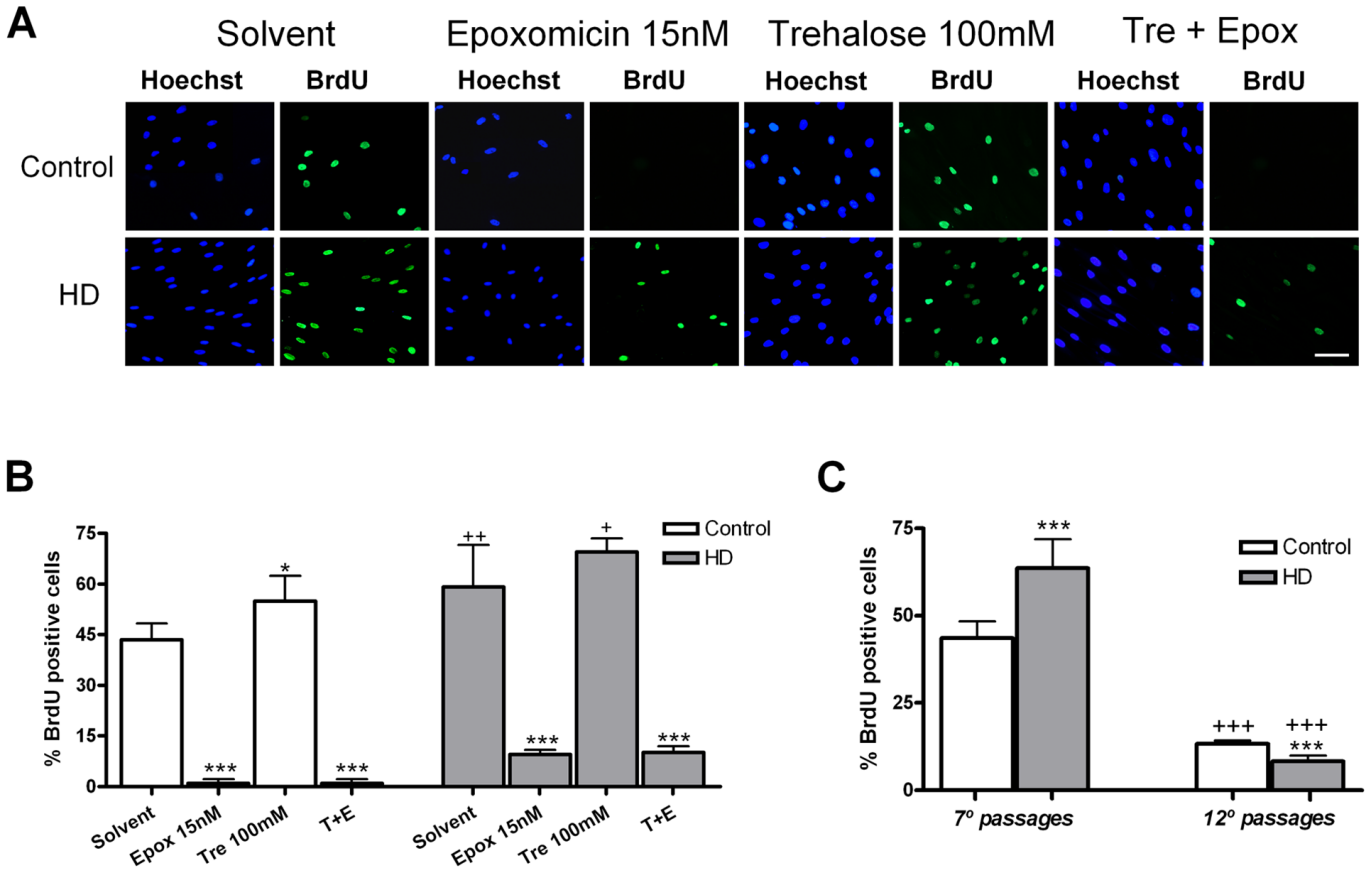
Effects of epoxomicin and trehalose in skin fibroblast cell cycle. (A) Photomicrographs of BrdU positive cells of dividing cells (green) and total nuclei stained with bis-benzimide (blue) from control and HD patients. (Scale bar  = 20 µm). (B) Percentage of BrdU positive cells with respect to the total number. (C) Comparison of the percentage of BrdU positive cells in early and late cell passage numbers. Values are expressed as the mean ± SD, *n* = 4 patients. Control cell number (mean per field) 33.80±1.417, *n* = 4. HD cell number (mean per field) 41.56±2.025, *n* = 4. The data of each patient was obtained using 4 replicates. Statistical analysis was performed by one-way ANOVA with repeated measures followed by Bonferroni multiple comparison test: *p<0.05, ***p<0.001 *vs* Solvent; +p<0.05, ++p<0.01, +++p<0.001 HD *vs* controls.

Trehalose 100 mM produced an increase in the number of BrdU+ fibroblasts but failed to modify the epoxomicin-induced reduction of cell division after 48 hours of treatment (FIG. 2A and 2B). The study of the cell cycle showed differences in the proliferative capacity between control and HD fibroblasts. Young HD fibroblasts had higher BrdU incorporation than aged fibroblasts that showed lower BrdU uptake in HD than in controls (FIG. 2C).

### Trehalose protects from increased ROS production induced by epoxomicin

**Figure 3:**
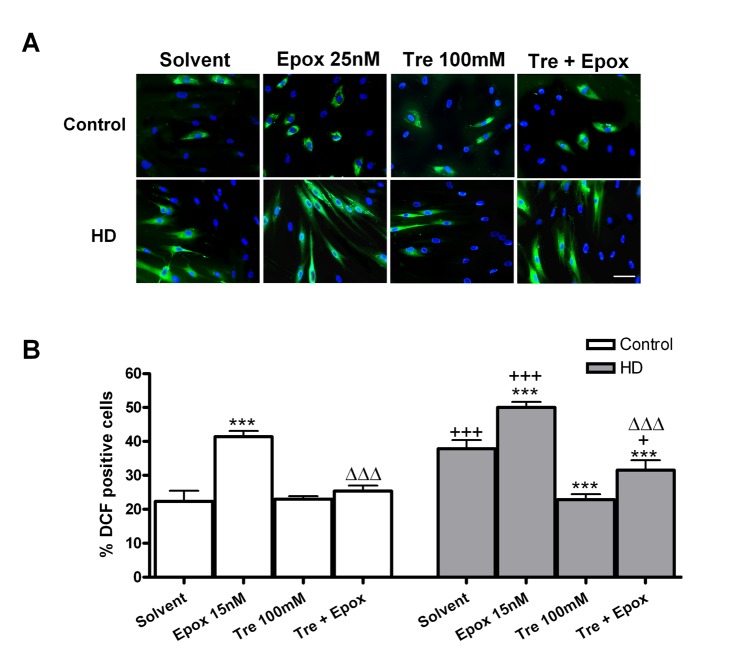
Epoxomicin increases ROS levels, which are reduced by trehalose in HD firboblasts. (A) 2′, 7′–dichlorofluorescin (DCF) immunocytochemistry (green) and total nuclei stained with bis-benzimide (blue) in control and HD fibroblasts and (B) percentage of DCF positive cells respect to the total number. (Scale bar  = 20 µm). Values are expressed as the mean ± SD, *n* = 4 patients. Control cell number (mean per field) 28.83±0.9280, *n* = 4. HD cell number (mean per field) 46.40±5.247, *n* = 4. The data of each patient was obtained using 4 replicates. Statistical analysis was performed by one-way ANOVA with repeated measures followed by Bonferroni multiple comparison test: ***p<0.001 *vs* solvent; +p<0.05, +++p<0.001 HD *vs* controls; ΔΔΔp<0.001 trehalose + epoxomicin *vs* epoxomicin.

HD fibroblasts showed higher ROS levels than controls, measured as DCF positive cells with respect to total cell number (FIG. 3). Epoxomicin increased ROS levels in both control and HD fibroblasts which were reduced totally with co-treatment with trehalose 100 mM. Furthermore, trehalose could reduce the basal level of ROS in HD fibroblasts. The photomicrographs were obtained with different camera settings (FIG. 3A) to obtain a similar fluorescence intensity, because HD fibroblasts had a much higher ROS fluorescence intensity.

### Trehalose protects against huntingtin and polyubiquitinated protein accumulation induced by epoxomicin in HD fibroblasts

**Figure 4:**
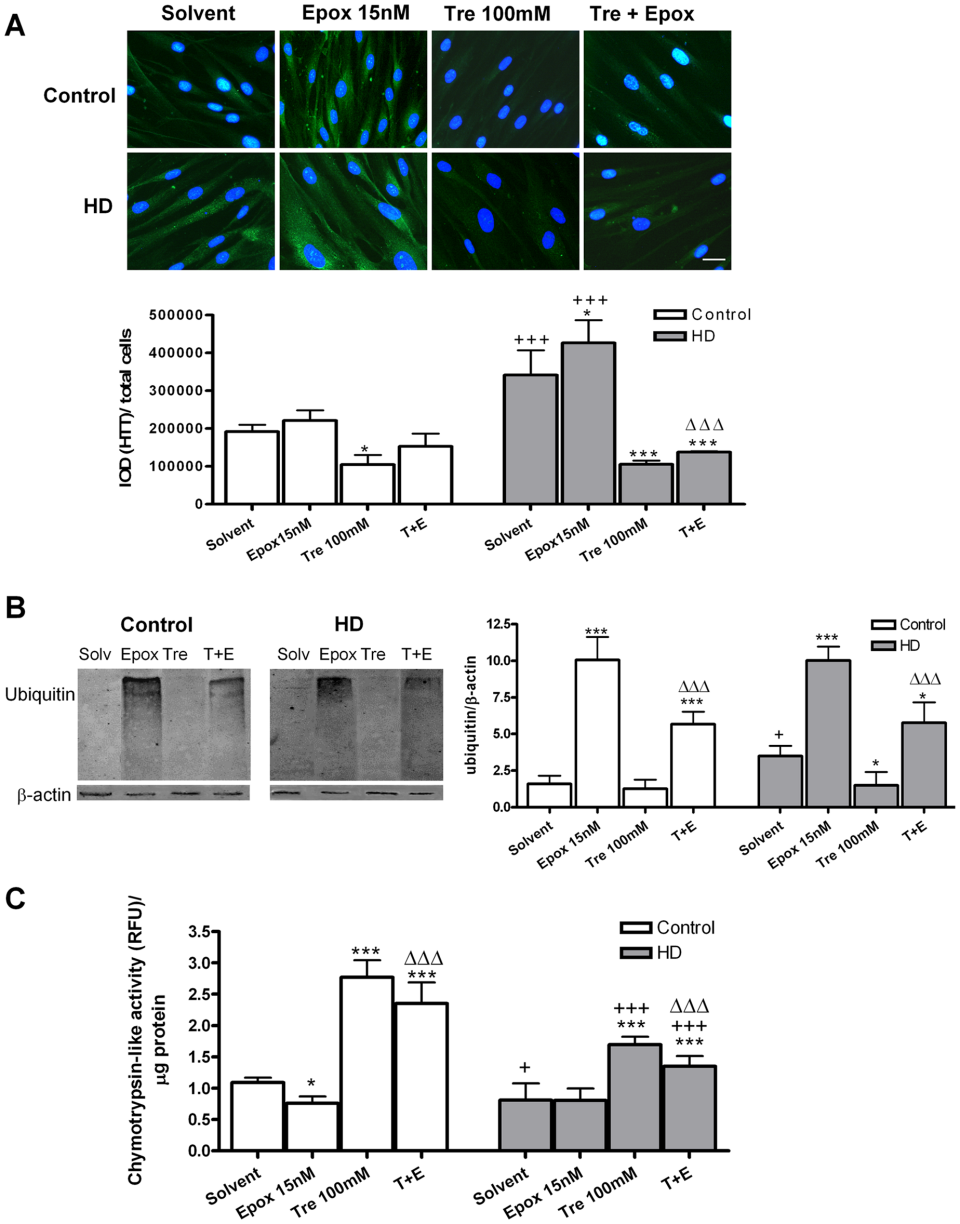
Trehalose protects against accumulation of Huntingtin and poly-ubiquitinated protein induced by epoxomicin and increases UPS activity. (A) Huntingtin immunocytochemistry (green) and total nuclei stained with bis-benzimide (blue). The histogram shows the ratio of Huntingtin (IOD) with respect to the total cell number. (Scale bar  = 20 µm) (B) Ubiquitinated protein accumulation and its corresponding densitometric analysis. (C) Chymotrypsin-like proteasome activity. Values are expressed as the mean ± SD, *n* = 4 patients. Control cell number (mean per field) 32,66±1.472, *n* = 4. HD cell number (mean per field) 44,67±0.3405, *n* = 4. The data of each patient was obtained using 4 replicates. Statistical analysis was performed by one-way ANOVA with repeated measures followed by Bonferroni multiple comparison test: *p<0.05, ***p<0.001 *vs* solvent; +p<0.05, +++p<0.001 HD *vs* controls; ΔΔΔp<0.001 trehalose + epoxomicin *vs* epoxomicin.

In untreated cultures, huntingtin (HTT) and polyubiquitinated protein levels were higher in HD fibroblasts than in controls. Treatment with epoxomicin induced the accumulation of these proteins (FIG. 4A and 4B). Trehalose reduced the levels of huntingtin below baseline in control and HD fibroblasts. In adition, trehalose reduced the levels of huntingtin in epoxomicin treated HD fibroblasts (FIG. 4A). Co-treatment with trehalose partially counteracted the effect of epoxomicin in polyubiquitinated proteins. As happened with ROS levels, trehalose reduceed the basal polyubiquitinated proteins in HD fibroblasts (FIG. 4B). With respect to the proteasome activity, the chymotrypsin-like activity is reduced in HD fibroblasts. Epoxomicin further reduced this activity, at least in controls, and trehalose increased this proteasomal activity even in cultures co-treated with epoxomicin (FIG. 4C).

### Effects of epoxomicin and trehalose on ERK-1/2 and HSP70 chaperone protein activation

**Figure 5:**
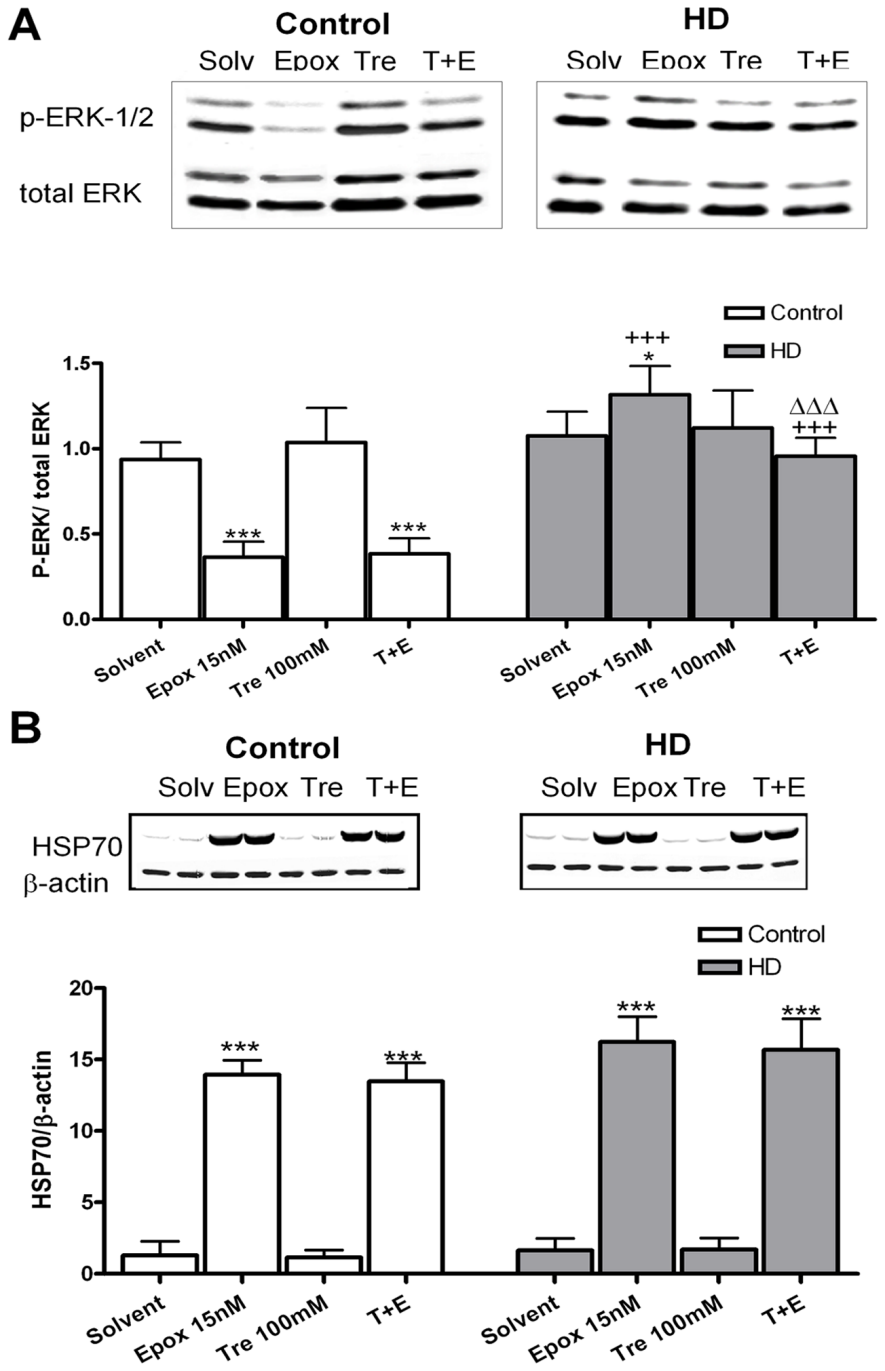
Effects of epoxomicin and trehalose on ERK-1/2 and HSP70 chaperone protein activation in HD fibroblasts. (A) Western blot of p-ERK-1/2 expression with regard to total ERK and its corresponding densitometric analysis in control and HD fibroblasts. (B) Western blot of HSP70 expression and its corresponding densitometric analysis. Values are expressed as the mean ± SD, *n* = 4 patients. The data of each patient was obtained using 4 replicates. Statistical analysis was performed by one-way ANOVA with repeated measures followed by Bonferroni multiple comparison test: *p<0.05, ***p<0.001 *vs* Solvent; +++p<0.001 HD *vs* controls, ΔΔΔp<0.001 trehalose + epoxomicin *vs* epoxomicin. There is an interaction between epoxomicin effect and genotype in ERK activation (F = 71.13 with a p value  = <0.0001). In HD, there is an interaction between the epoxomicin and trehalose effects in ERK activation (F = 12.67 with a p value  = 0.0013).

After 24 hours of treatment, epoxomicin reduced levels of phosphorilated ERK and trehalose could not revert it in controls. In HD fibroblasts, epoxomicin increased p-ERK levels. Co-treatment with trehalose reverted the effects of the epoxomicin on p-ERK levels in HD fibroblasts (FIG. 5A). Epoxomicin greatly increased HSP70 levelsand this effect was not reverted by trehalose. (FIG. 5B).

### Effects of trehalose in macroautophagy and chaperone-mediated authohpagy

**Figure 6:**
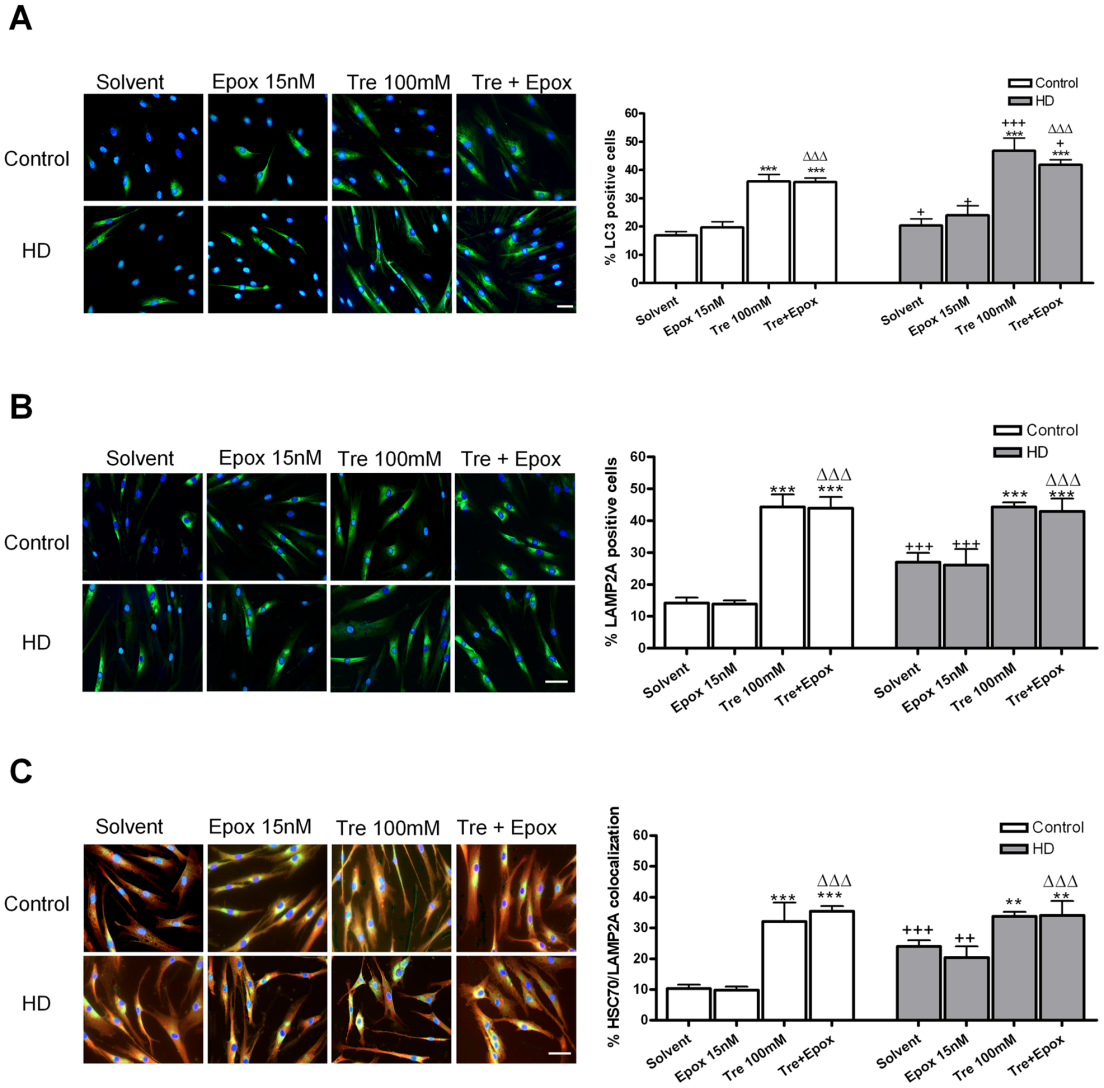
Macroautophagyc pathway and chaperone-mediated autophagy in control and HD human skin fibroblasts. (A) LC3 immunocytochemistry (green) and total nuclei stained with bis-benzimide (blue) and the percentage of LC3 positive cells with respect to the total cell number. (B) LAMP2A immunocytochemistry (green) and total nuclei stained with bis-benzimide (blue) and percentage of LAMP2A positive cells. (C) LAMP2A (green) and HSC70 (red) colocalization (yellow) and percentage of LAMP2A and HSC70 colocalization respect to the total cell number. (Scale bar  = 20 µm). Values are expressed as the mean ± SD, *n* = 4 patients. Control cell number (mean per field) 30.94±2.012, *n* = 4. HD cell number (mean per field) 46.89±4.587, *n* = 4. The data of each patient was obtained using 4 replicates. Statistical analysis was performed by one-way ANOVA with repeated measures followed by Bonferroni multiple comparison test: **p<0.01, ***p<0.001 *vs* Solvent; +<0.05, +p<0.05, ++p<0.01, +++p<0.001 HD *vs* controls; ΔΔΔp<0.001 trehalose + epoxomicin *vs* epoxomicin.

The LC3 expression was upregulated in HD fibroblasts and trehalose produced a twofold increase even when epoxomicin is present in both controls and HD fibroblasts (FIG. 6A). As happened with LC3 levels, LAMP2-A levels were higher in HD fibroblasts, possibly, as a compensatory response for decreased UPS activity and ubiquitinated protein accumulation. Trehalose considerably increased LAMP2-A expression with or without epoxomicin treatment (FIG. 6B). HSC70 can colocalizate with LAMP2-A to participate in chaperone-mediated autophagy (CMA) [15]. We showed the colocalization between these two proteins (FIG. 6C) which indicated that trehalose also increases CMA.

## Discussion

We have shown, in this study, that epoxomicin produceed a differential dose-dependent increase in activation of caspase-3 in control and HD fibroblasts. Epoxomicin also increaseed ROS, ubiquitinated proteins and huntingtin accumulation in HD fibroblasts. All these changes caused by this proteasome inhibitor are reverted by trehalose. Our experiments have revealed basal differences between HD and control fibroblasts. HD fibroblasts had higher levels of BrdU incorporation at early passage numbers. However, when we studied the proliferative rate at late passage numbers, BrdU incorporation was diminished, to a great extent in HD fibroblasts. These results suggested that HD fibroblasts suffered a faster evolution to a senescent phenotype, probably caused by their deficiency in the UPS activity [21]–[23]. The treatment with epoxomicin for 48 hours stopped replication 99% in control and 85% in HD fibroblasts. Furthermore, trehalose increased the replication in both HD and control fibroblasts but could not prevent the cell cycle arrest produced by epoxomicin. UPS is implicated in cell cycle [16], [17] and has also shown a decreased UPS activity in senescent cells with lower replicative capacity [24], which is consistent with the cell cycle arrest caused by epoxomicin. However, we observed that HD fibroblasts had a higher replication rate than controls as described by Goetz et al [25].

Proteasomal dysfunction has been considered a mechanism of production of neurodegenerative disorders including Huntington's disease. Huntingtin protein is degraded by the ubiquitin-proteasome system and autophagy [26], [27]. Abnormal UPS function has been found in HD brain and skin fibroblasts previously [28], [29]. We have shown that HD fibroblasts had high levels of ubiquitinated proteins and huntingtin. We showed in this study that trehalose, as an autophagy enhancer, compensated the lack of activity in the UPS, even in fibroblasts treated with epoxomicin in both genotypes.

In HD there is abnormal mitochondrial activity and excessive production of free radicals [30]–[33]. Reduction of respiratory activity in HD has been found in brain but not in fibroblasts [28], [34], [35]. We found that HD skin fibroblasts had an increased level of ROS and a diminished chymotrypsin-like activity (UPS) with respect to controls, and these effects are reverted by trehalose. In controls, trehalose also increased UPS and reversed epoxomicin-induced ROS increase. Some studies report a relation between UPS dysfunction, ROS production and mitochondrial impairment [35]–[37]. Increasing autophagy using trehalose improved UPS activity by mutant huntingtin elimination which could alleviate the mitochondrial impairment and reduce ROS formation.

ERK proteins belong to the MAP kinase family and are related to promoting cell survival. However, the effects of the activation of this kinase are dependent on the intensity and the maintenance of the active state. Persistent and strong activation of ERK leads to cell death [38], [39], whereas a short-lived activation of ERK is associated with survival [21], [40]. Furthermore, a downregulation of ERK is related to an inhibition of cell proliferation [22], [23]. In our experiments we showed that ERK was downregulated in presence of epoxomicin in control fibroblasts, which is consistent with the cell cycle arrest observed in the BrdU assay. The situation was completely different in HD fibroblasts. Epoxomicin induced an ERK activation of 22% in 24 hours resulting in promotion of cell death, which is reverted with the treatment with trehalose.

HSP70 functions as a chaperone and protects neurons from protein aggregation and toxicity binding unfolded or partially folded proteins [41]. Members of the HSP40 and HSP70 chaperone families have also been found to colocalize with nuclear aggregates in several polyQ diseases, both in human and mouse brains [42], [43]. In our experiments, we appreciated a huge increase in HSP70 levels when skin fibroblasts were treated with epoxomicin in both control and HD. This fact implies a protective role of HSP70 against the proteasome inhibitor effects, among which is included protein accumulation.

UPS inhibition may induce neurodegeneration and it could be reverted by increasing autophagy [44]. LAMP-2A is a Lysosome Membrane Protein so its expression means also lysosomal activation, and LC3 is localized in the inner and outer membrane of autophagosomes [45]. We have observed an increase in autophagy, using LC3 and LAMP2-A markers, in HD and control fibroblasts treated with trehalose.

Due to the beneficial effects of trehalose in proteinopathies, as an autophagy enhancer, chemical chaperone and antioxidant, as well as its virtual absence of toxic effects even at high doses, this disaccharide is an interesting candidate for testing in clinical trials in HD patients.
